# 622. Population Pharmacokinetic Analysis and Probability of Target Attainment Simulations of Pivmecillinam for the Treatment of Uncomplicated Urinary Tract Infection

**DOI:** 10.1093/ofid/ofac492.674

**Published:** 2022-12-15

**Authors:** Anne Laurence Santerre Henriksen, Maxime Lagraauw, Marita Prohn, Lars Lindbom

**Affiliations:** Maxel Consulting ApS, Jyllinge, Sjelland, Denmark; qPharmetra, Nijmegen, Gelderland, Netherlands; qPharmetra, Nijmegen, Gelderland, Netherlands; qPharmetra, Nijmegen, Gelderland, Netherlands

## Abstract

**Background:**

A population pharmacokinetic model was developed to characterize mecillinam (MEC) pharmacokinetics (PK) and urine exposure after intravenous (IV) administration of MEC or after oral (PO) administration of its prodrug pivmecillinam (PIV) in healthy subjects and patients with renal impairment (RI) or infections. MEC is a β-lactam antibiotic with a targeted spectrum of activity against Enterobacterales. The model was used to investigate various PIV treatment regimens and covariate scenarios on plasma exposure and urine excretion, and to perform probability of target attainment (PTA) simulations in support of dose justification for the treatment of uncomplicated urinary tract infection (uUTI).

**Methods:**

The analysis was based on MEC PK data obtained in plasma, serum, and urine in 15 clinical studies. The dataset included a total of 3964 plasma or serum concentrations and 989 urine samples obtained in 228 subjects. Those 228 subjects consisted of 172 healthy volunteers, 23 patients with infections (uUTI, Gram-negative infection, typhoid, or paratyphoid fever), and 33 patients with various degrees of renal impairment (RI). Subjects were treated with single or multiple doses of MEC (IV, 200-1410 mg) or PIV (PO, 137-500 mg).

**Results:**

MEC PK profiles in plasma and urine were well characterized by a 2-compartment distribution model with first-order renal elimination and nonlinear non-renal elimination. Oral absorption of PIV was best described using a single (Erlang) transit compartment. The PK model included parameter-covariate relationships for WT on all clearance and volume parameters (with fixed allometric exponents of 0.75 and 1), a nonlinear dose effect on bioavailability, formulation effects on Ka, food effects on both Ka and F1, and effects of renal function on both CL and Km. PTA simulations for the proposed dosing regimen of 200 mg TID predicts up to 70% fT*>*MIC for the CLSI susceptibility breakpoint of 8 mg/L in 90% of the population for a voiding frequency of 45 min.

**Conclusion:**

MEC PK was well characterized by a 2-compartment distribution model with first-order renal and nonlinear non-renal elimination. The oral PIV absorption was best described using a single transit compartment. PTA simulations were supportive of a 200 mg TID dosing.

**Disclosures:**

**Anne Laurence Santerre Henriksen, PhD**, Shionogi: Contractor|UTILITY therapeutics Ltd: Advisor/Consultant **Maxime Lagraauw, PhD**, qPharmetra: Employee **Marita Prohn, n/a**, qPharmetra: Employee **Lars Lindbom, PhD**, qPharmetra: Employee.

